# Obesity as a form of malnutrition: over-nutrition on the Uganda “malnutrition” agenda

**DOI:** 10.11604/pamj.2017.28.49.11176

**Published:** 2017-09-20

**Authors:** Christine Ngaruiya, Alison Hayward, Lori Post, Hani Mowafi

**Affiliations:** 1 Department of Emergency Medicine, Yale School of Medicine, New Haven, CT, USA

**Keywords:** Uganda, East Africa, Africa, nutrition, malnutrition, overweight, obesity

## Abstract

The objectives were to highlight the burden of overweight and obesity as an additional area of importance for the malnutrition agenda in Uganda and to provide evidence-based considerations for stakeholders involved. Introduction: Mirroring other Low- and Middle-Income Countries (LMICs), Uganda is experiencing a “double burden” of over-nutrition related issues - both obesity and overweight, and related non-communicable diseases (NCDs) alongside the under-nutrition that has long plagued the country. Despite the commonplace assumption that under-nutrition is the predominant form of malnutrition in Uganda, we explore recent literature that in fact, challenges this notion. While food insecurity has contributed to the under-nutrition problem, a lack of dietary diversity also has a demonstrated role in increasing over-nutrition. We cannot afford to ignore over-nutrition concomitant with stunting and wasting in the country. Increase in the burden of this less acknowledged form of malnutrition in Uganda is critical to investigate, and yet poorly understood. A move towards increased regionally targeted over-nutrition research, funding, government prioritization and advocacy is needed.

## Essay

Mirroring other Low- and Middle-Income Countries (LMICs), Uganda is experiencing a “double burden” of over-nutrition related issues – both obesity and overweight, and related non-communicable diseases (NCDs) alongside the undernutrition that has long plagued the country [1–3]. Despite the commonplace assumption that under-nutrition is the predominant form of malnutrition in Uganda, recent literature, in fact, challenges this notion. Raschke and Popkin have demonstrated patterns in nutritional transition in other developing countries like China and India undergoing epidemiologic and economic transition, a trend that appears to be prominent in Uganda at this time as well [4, 5]. While food insecurity has contributed to the under-nutrition problem, a lack of dietary diversity also has a demonstrated role in increasing over-nutrition. We cannot afford to ignore over-nutrition concomitant with stunting and wasting in the country [6]. Increase in the burden of this less acknowledged form of malnutrition in Uganda is critical to investigate, and yet poorly understood [7, 8]. A move towards increased regionally targeted over-nutrition research, funding, government prioritization and advocacy is needed.

## Research

**Figure 1:**
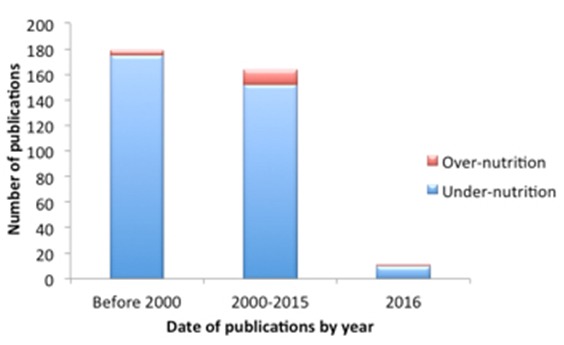
Comparison of total number of pubmed publications using search terms “undernutrition and Uganda” and “Overnutrition and Uganda” (search completed April 2016)

While a handful of studies have contributed glimpses into the prevalence of over-nutrition in Uganda, there is a paucity of data on the underlying factors, which limits comprehensive understanding of the increasing pandemic of obesity and chronic disease. No systematic review on malnutrition and chronic disease exists for Uganda. There is a dearth of scholarly inquiry related to over-nutrition in Uganda in stark contrast to under-nutrition. A search in the United States Department of Agriculture National Agricultural Library PubAg database using search terms “malnutrition and Uganda”, revealed twelve out of thirteen articles used the term “malnutrition” to describe issues of under-nutrition. A single systematic review article by Raschke et al, further discussed later in this paper, highlighted the “nutrition transition” [9]. Furthermore, as evidenced by a Pubmed search, over the past decade, there has been a slight increase in the number of over-nutrition research publications in Uganda, but not with the same zeal as is under-nutrition. There are tenfold more studies on under-nutrition compared to over-nutrition (Figure 1). Epidemiological studies demonstrate an increasing burden of over-nutrition compared to under-nutrition. The latest Uganda Demographic Health Survey (DHS), published in 2011, documents increases in obesity over the last decade [10, 11]. Uganda simultaneously experienced a decreased prevalence of underweight children less than five years old from 2006 to 2011 (16% to 14%) [10, 11]. Mendez et al. (2005) found overweight Ugandan women as high as 23% in the urban areas, almost four-fold the prevalence of underweight, and 9% in rural areas [12]. A 2013 study confirmed the DHS, revealing that in non-pregnant mothers in rural southwestern Uganda, rates of overweight, measured by BMI, were more than triple compared to the rates of underweight counterparts [13]. Certain regional and sub-population studies in specialty clinics have limited evidence of obesity and its negative sequelae. Muyinga et al. (2016) found 58% of patients at an HIV clinic had metabolic syndrome including a waist circumference indicative of excess weight. Several other HIV-clinic based studies support this concerning trend [14, 15]. Overweight status was 42% in a stroke risk factors study in an urban population [8]. The WHO STEPS tool used in two regions of Uganda also found nearly half of the respondents were overweight [16]. A handful of studies have begun to scratch the surface of the epidemiology of over-nutrition in Uganda. Turi’s et al. (2011) analysis of UDHS spatial distribution of overweight and obesity was most evident in southwestern parts of Uganda [17]. A multi-regression analysis of LMICs (including Uganda) conducted by Jones-Smith et al. in 2010 demonstrated a one percent increase in obesity per wealth quintile [18].

### Areas of specific research need in Uganda

There is some evidence that food insecurity contributes to malnutrition [6]. Our Ugandan research revealed high-carbohydrate meals, such as “posho”, a staple porridge made from starchy flours like cormeal or cassava flour, were common. When possible, this is served with meat, which not only contributes to satiety, but culturally is viewed as better or associated with higher class status than beans, “greens” or vegetables – resulting in increased caloric intake but frequently deficient in micro-nutrients. This lack of dietary diversity results in under-nourished children whose development is stunted and over-nourished adults prone to obesity and insulin resistance. These and other cultural drivers for food diversity and food preference need further investigation. Cultural misconceptions need to be corrected to improve the public’s health. Furthermore, while foundational research has shown that childhood dietary diversity is a contributory factor for obesity, however, there are little to no over-nutrition studies of Ugandan children [19–21]. Christoph et al. reported a 5% prevalence of over-nutrition related disease in a sample of 151 students (as compared to 10% prevalence of under-nutrition) in 2014 [22]. Peltzer et al. studying school children in Uganda and Ghana, found that while cigarette use was associated with overweight or obesity, being sedentary was not [23]. Such findings suggest that risk factors must be specific to Uganda.

## Policy and funding

A better understanding of the over-nutrition problem is important to guide policy-makers, translational or implementation research, and interventions to address the problem. Funders, however, may be undermining success. Holdsworth et al. found that while Africa-based stakeholders, including researchers and governments, ranked the study of ecological nutrition – influence of environment on nutrition - highly, funders discounted this [24]. To that end, we need to align research agendas with social problems. Some might argue that the rise of over-nutrition is a marker of success in the war on food insecurity and that countries can delay addressing such problems until they reach targets for economic development and the elimination of hunger. Such an approach is short-sighted and would have devastating health and economic impacts for low- and middle-income countries. Over-nutrition and associated non-communicable diseases are conditions that require expensive, life-long therapies and investments in therapeutic care that these countries are currently ill-equipped to make. Over-nutrition and related chronic disease cause premature mortality, affecting the most economically productive members of the society. Premature losses of breadwinners have a domino effect leading to lost income, increased poverty and adverse health outcomes [1]. Policy addressing these problems early on through preventative measures is needed at the Ministry of Health level, the district level, the institutional level and amongst community government. Uganda has made a political commitment to optimize nutrition at the national level however with a primary focus on under-nutrition. The Uganda Nutrition Action Plan (UNAP), a five year national framework outlining priority targets and strategies to address malnutrition is entirely focused on problems stemming from under-nutrition and makes no mention of addressing the over-nutrition problems in the country [25]. Along with the UNAP, Uganda has joined the global Scaling Up Nutrition movement, comprised of 55 countries working together to improve nutrition using multi-sectoral strategies that engage stakeholders at many different levels to achieve World Health Assembly goals, which also addresses under-nutrition. In 2015, the country launched the inaugural Uganda Multi-sectoral Food Security and Nutrition Project, a World Bank funded project, to address malnutrition issues, through emphasizing multi-sectoral involvement in planning, implementing nutrition interventions, and strengthening capacity and research to support these interventions [26]. The US government, through the USAID Feed the Future program, has also implemented strategies to address targets of hunger and poverty. This program acts on processes all the way from policy development to provision of technical assistance to farmers, and enforcement of appropriate clinical care for under-nutrition management. These programs’ budgets are tens of millions of dollars and are currently evaluating their early performance. These are just a sample of the numerous funding ventures all dedicated to addressing under-nutrition, however no such prioritization in policy or funding strategies are given to over-nutrition. It is imperative that such programs have plans in place not only to address the nutritional challenges of yesterday and today (stunting and wasting) but also to create strategies to combat the nutritional challenges of tomorrow (nutritional transition, overweight and NCDs).

## Advocacy

Strategies to target and affect over-nutrition and related illnesses should be made an imminent priority. Ministries and development partners in Uganda continue to focus on the under-weight threats to public health. Addressing problems on opposite sides of the weight spectrum is not an “either/ or” proposition. Increased advocacy at the government level is needed to drive the move towards incorporating over-nutrition at the forefront of the malnutrition agenda. A variety of well-established intervention programs with a focus on over-nutrition have the potential to be successful in Uganda. Sites for active enforcement and further advocacy at the institutional level include optimizing existing HIV chronic care structures for institution of over-nutrition management and control [27]. Additionally, global trade-driven production, increased consumption of imported foods and advertising have all been postulated as contributors to an increasing burden of over-nutrition and cannot be ignored [5]. As such, enforcement of the use of nutrition labels and rules of marketing including sensitivity towards certain vulnerable groups such as children should take place. Finally, school education programs are a fundamental and potentially affordable strategy. Targeting the youth, where the earliest food choices are made and the onset of chronic disease occurs, will give programs the opportunity for the greatest payoff.

Addressing the double burdens of under- and over-nutrition will be one of the primary public health challenges for Uganda in the decades to come. Balancing these priorities and developing economic, agricultural, nutritional and public health policies that support a balanced agenda will be essential to improving outcomes in all these domains. Uganda and its development partners must develop strategies to construct the evidence base upon which to develop new policies, identify effective interventions, and to integrate such evidence and interventions into their development plans in the future.

## Competing interests

The authors declare no competing interest.
